# Bacterial Cellulose-Derived Biochar for Electrochemically Assisted Fenton Degradation of Methylene Blue

**DOI:** 10.1021/acsomega.5c02022

**Published:** 2025-06-16

**Authors:** Gladston L. dos Santos, Rebeca E. S. Barros, Yslaine A. de Almeida, Katlin I. B. Eguiluz, Giancarlo R. Salazar-Banda, Iara F. Gimenez

**Affiliations:** † Programa de Pós-graduação em Química, Universidade Federal de Sergipe, São Cristovão, Sergipe 49100-000 Brazil; ‡ Laboratório de Eletroquímica e Nanotecnologia − LEN, Instituto de Tecnologia e Pesquisa − ITP, Aracaju, Sergipe 49032−490 Brazil; § Programa de Pós-graduação em Engenharia de Processos, Universidade Tiradentes − UNIT, Aracaju, Sergipe 49032−490 Brazil

## Abstract

This study explores the potential of biochar derived from bacterial cellulose (BC-derived biochar) as a catalytic support in Fenton-type processes for the degradation of methylene blue (MB) in aqueous solutions. The biochar was synthesized by modifying purified BC membranes with Fe^2+^/Fe^3+^ ions, followed by calcination at 400 °C. The performance of three treatment approaches (electrochemical degradation, heterogeneous Fenton process, and a combined treatment integrating both methods) was evaluated. The results highlighted the superior efficiency of the combined treatment, demonstrating its potential for practical applications. A 2^3^ factorial design with a central point was employed to evaluate the effects of key variables in the electrochemically assisted Fenton process: MB concentration, BC-derived biochar concentration, and reaction pH. Statistical analysis, with an *R* ^2^ value of 0.989, revealed that only the dye concentration and biochar concentration had statistically significant effects, while pH and interaction effects were negligible. Discoloration efficiencies reached nearly 80% for 10 ppm solutions at both acidic and basic pH, with a maximum treatment time of 60 min, demonstrating the versatility and effectiveness of the Fenton system in diverse media. Additionally, a kinetic study further supported these findings, with the best result fitting a first-order reaction model (*R* ^2^ = 0.984). The total organic carbon analysis confirmed the method’s efficiency, showing a reduction in the organic compound by over 50% for both 10 and 55 ppm solutions. These results demonstrate that the proposed method is a promising alternative for treating MB dye and can be extended to the degradation of other organic compounds.

## Graphical abstract

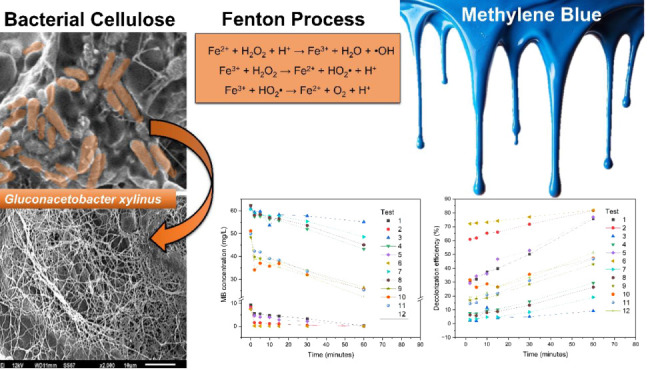

## 1 Introduction

The textile industry plays a fundamental role in the global economy and daily life by transforming fibers into fabrics and garments. However, it is also a significant source of environmental pollution, particularly due to the release of wastewater containing synthetic dyes.^1–3^ Among these dyes, methylene blue (MB) is one of the most commonly used in dyeing processes. It is also employed in diagnostic and therapeutic applications, as well as in the food industry. Despite its versatility, prolonged exposure to MB poses serious health risks such as skin irritation, neurological disturbances, and cardiovascular disorders.^5,6^

Various treatment methods have been explored to remove dyes from industrial effluents, including adsorption, bioremediation, and photocatalysis.^7^ Among these, advanced oxidation processes (AOPs) stand out due to their ability to generate powerful oxidizing species capable of degrading a wide range of organic pollutants.^8^ The Fenton reaction, in particular, is highly regarded for its efficiency and environmental compatibility.^9,10^ However, the classical homogeneous Fenton process suffers from limitations, such as the need for an acidic pH to avoid iron precipitation and the generation of excess iron residues in treated water.

In response to these limitations, heterogeneous Fenton systems have emerged as a promising alternative. They operate effectively across broader pH ranges and allow for the recovery and reuse of iron-based catalysts, enhancing both the sustainability and cost-efficiency of the treatment.^4^ The key reactions involved in the Fenton process are

1 $${\mathrm{Fe}}^{2+}{+H}_{2}O_{2}{+H}^{+}\to {\mathrm{Fe}}^{3+}{+H}_{2}O+\mathrm{•OH}$$

2 $${\mathrm{Fe}}^{3+}+H_{2}O_{2}\to {\mathrm{Fe}}^{2+}+{\mathrm{HO}}_{2}•+H^{+}$$

3 $${\mathrm{Fe}}^{3+}+{\mathrm{HO}}_{2}•\to {\mathrm{Fe}}^{2+}+O_{2}+H^{+}$$

Recently, research has focused on developing new catalysts to improve Fenton-type processes. Bacterial cellulose (BC), produced by microorganisms such as *Gluconacetobacter xylinus*, has drawn attention due to its abundance, renewability, and structural properties such as high porosity, crystallinity, and specific surface area.^10,11^ These features make BC an attractive precursor for producing biochar, particularly when it is impregnated with Fe^2+^/Fe^3+^ ions and subjected to calcination.

Despite recent advances, the integration of biochar derived from biomass carbon (BC) with electrochemical assistance in heterogeneous Fenton systems remains underexplored. Moreover, few studies employ robust experimental designs or provide comprehensive analyses of reaction kinetics and mineralization efficiency.

Therefore, this study proposes the use of bacterial cellulose-derived biochar (KBPFC) as a catalytic support in an electrochemically assisted heterogeneous Fenton system for degrading methylene blue. The system’s efficiency was assessed through comparative performance tests, a 2^3^ factorial design with a central point, kinetic modeling, and total organic carbon (TOC) analysis, aiming to evaluate its applicability under different dye concentrations, catalyst loads, and pH levels.

## 2 Materials and Methods

### 2.1 Reagents

The following reagents were used in this study: methylene blue (MB, Neon), ferrous chloride tetrahydrate (FeCl_2_·4H_2_O, Sigma-Aldrich, analytical grade), ferric chloride hexahydrate (FeCl_3_·6H_2_O, ACS Científica, analytical grade), hydrochloric acid (HCl, 37%, Dinâmica, analytical grade), sodium hydroxide (NaOH, VETEC, analytical grade), sodium azide (Dinamica), potassium iodide (Synth), *tert*-butyl alcohol (ACS), and hydrogen peroxide (H_2_O_2_, 35%, Neon, analytical grade). All chemicals were used as received without further purification.

### 2.2 Production of Bacterial Cellulose (BC) from Kombucha

The production of BC followed the methodology described by Sederavičiu̅tė et al.^12^ Briefly, the culture medium was prepared by adding 4 g of green tea to 1 L of boiling water, followed by a 15 min resting period to allow infusion. Then, 100 g of sucrose, 100 mL of kombucha extract, and the “mother” SCOBY were added to the mixture. The culture was then maintained under static conditions for 14 days at room temperature (25 °C), during which the BC pellicle (referred to as the “daughter” SCOBY) was formed.

### 2.3 Purification of Bacterial Cellulose Film

Biofilms were purified according to the methodology reported by Sederavičiu̅tė et al.,^12^ which involved immersing the film in 0.1 M NaOH for 24 h, with the medium exchanged at 1, 2, and 4 h intervals. Subsequently, the films were rinsed with 2 L of distilled water and stored under refrigeration.

### 2.4 Preparation of Catalytic Support for the Heterogeneous Fenton-Type Reaction

Deposition of Fe^2+^/Fe^3+^ on the biofilm was carried out according to the method reported by Wibowo et al.^4^ BC films were immersed in precursor FeCl_2_ and FeCl_3_ solutions containing 0.01 mol L^–1^ Fe^2+^ and 0.02 mol L^–1^ FeCl_3_ for 24 h at room temperature (25 °C), acquiring an ocher color (Figure 1a). Pellicles with two different masses (15 and 20 g) were immersed in the iron-containing solution, and the reduction in Fe concentration in the solutions was analyzed through ICP-OES to quantify the amount of iron deposited on the BC. After this step, the film was immersed in 4 mol L^–1^ NaOH at 60 °C for 15 min, during which the pellicles turned brown (Figure 1b). The film was then thoroughly rinsed with 2 L of distilled water to remove residual impurities and stored under refrigeration for subsequent use. After deposition, the resulting film was calcined in an EDG 3000 muffle furnace under an air atmosphere. The calcination process involved heating the sample to 400 °C at a rate of 10 °C/min, followed by a dwell time of 5 min. This procedure yielded a dark brown powder (Figure 1c), which was subsequently used for further characterization and application.

**Figure 1:**
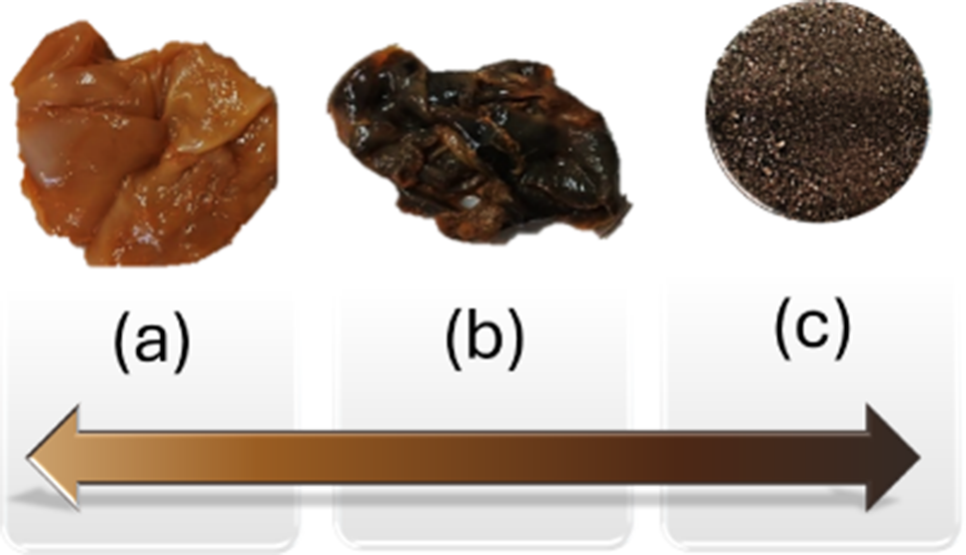
Photographs of (a) BC after 24 h immersed in FeCl_2_/FeCl_3_; (b) pellicle shown in (a) after immersion in 4 mol L^–1^ NaOH; (c) biochar obtained after calcination at 400 °C.

Before characterization and application, the samples were frozen for 60 min in a Liotop Ultrafreezer (model UFR30) and subsequently lyophilized for 24 h using a Liotop Lyophilizer (model L101) to ensure complete drying. The unpurified BC samples were designated as KBSP, while those purified with NaOH were labeled KBP. Samples that underwent purification followed by Fe^2+/^Fe^3+^ ion deposition were identified as KPPF, whereas those further subjected to calcination were referred to as KBPFC, as shown in Table 1.

**Table 1:** Sample Codes and Their Meanings

Code	Identification
**KBSP**	Untreated kombucha film
**KBP**	Film purified with NaOH
**KBPF**	Postdeposition film of Fe^2+/^Fe^3+^ ions
**KBPFC**	Postdeposition and calcination film

### 2.5 Characterization of Samples

X-ray diffraction (XRD) was measured using an XRD-7000 Shimadzu instrument with CuKα radiation, operating at 40.0 kV voltage and 30.0 mA current, in a continuous scan mode with a scanning speed of 2.0 deg min^– 1^, a sampling pitch of 0.0200°, and a preset time of 0.60 s. Fourier transform infrared spectra (FTIR) were obtained using an FTIR Spectrum Two spectrometer from PerkinElmer, analyzing KBr pellets in the range of 4000 cm^–1^ to 450 cm^–1^, a resolution of 4 cm^–1^, and 20 scans per sample. For thermogravimetric (TG) analysis, the equipment used was the Shimadzu model TGA-50, applying a controlled temperature program ranging from 30 to 1000 °C on a platinum sample holder, with a heating rate of 10 °C min^– 1^, an inert gas (N_2_) flow rate of 100 mL min^– 1^, and a sample mass of approximately 5 mg. Inductively coupled plasma optical emission spectrometry (ICP/OES) analysis was performed to verify the variation in iron content in the solution before and after deposition on the films (KBPF). The equipment used was the VARIAN model axial 720-ES. Scanning electron microscopy (SEM) images were acquired using a JEOL microscope (JSM-6510LV) operating at an accelerating voltage between 10 and 20 kV. To enhance image contrast, the samples were coated with silver (Ag) using a Kurt J. Lesker Company (180) Sputter Coater/Evaporator. For the purified and modified samples, an energy-dispersive spectroscopy (EDS) scan was performed to confirm the deposition on the material. For elemental determination, a Shimadzu Ray Ny EDX-720 energy-dispersive X-ray fluorescence spectrometer was used. The voltage applied to the X-ray tube was set to 15 and 50 keV, with a current of 100 μA, a detector dead time of 40%, and a 10 mm collimator. Spectra were sequentially acquired in the range of 0 to 40 keV, with an irradiation time of 100 s.

The specific surface areas of the materials were assessed via nitrogen adsorption–desorption isotherms, applying the Brunauer–Emmett–Teller (BET) theory. Microporous characteristics were evaluated using the Dubinin–Radushkevich (DR) approach, while pore size distribution and diameters were analyzed through density functional theory (DFT), employing a Quantachrome Nova 1200 porosimeter with nitrogen as the adsorbate.

### 2.6 Treatment of MB-Containing Solutions: Electrochemical System, Fenton System, and Electrochemically Assisted Fenton System

All electrochemical characterizations were performed in a 0.06 M NaCl solution with the aid of a potentiostat/galvanostat from Autolab PGSTAT302N (Metrohm), connected to a conventional two-electrode cell with a 70 mL capacity. The working electrode employed for electrochemical oxidation was a mixed metal oxide anode, specifically Ti/(RuO_2_)_0.5_(IrO_2_)_0.5_, with a geometric area of 6 cm^2^. The electrode was previously prepared using the Pechini method, followed by calcination via hybrid microwave heating at a final temperature of 350 °C.^13^ A titanium plate with a geometric area of 5.6 cm^2^ served as the counter electrode. The scheme of the electrochemical system used to treat MB can be seen in Figure S1.

For comparison purposes, three initial experiments were carried out: the first using only the electrochemical system, the second using only the addition of the calcined film KBPFC (0.06 g, L^– 1^ = 42 mg) and 5 drops of 35% H_2_O_2_ (approximately 0.25 mL) for the heterogeneous Fenton reaction. The last experiment was carried out using both the electrochemical system and KBPFC-Fenton. All experiments had a fixed volume of 70 mL, with stirring at 300 rpm, and for the electrochemical system, the current density was set at 10 mA, MB concentration at 10 ppm, and pH 6. The experiment was performed in duplicate, and the values were analyzed by using a UV–vis spectrophotometer.

In order to evaluate the nature of generated reactive oxygen species during the catalytic degradation of MB, experiments were carried out in the presence of quenchers, including sodium azide (quencher of singlet oxygen), *tert*-butyl alcohol, and potassium iodide (quenchers of hydroxyl radicals).^14^ Analogous experiments were performed in the absence (control) and presence of quenchers as follows. KBPFC was added to yield a concentration of 0.6 g L^– 1^ to 70 mL of 10 ppm MB solutions, with the pH adjusted to approximately 3 to favor the Fenton reaction. Subsequently, five drops of 35% H_2_O_2_ (approximately 0.25 mL) were added to the reaction mixture, which was then stirred for 30 min prior to UV/visible analysis. To investigate the effect of H_2_O_2_ concentration, control experiments were conducted under identical conditions, varying the number of drops of 35% H_2_O_2_ to 1, 3, and 5.

Next, a 2^3^ factorial design with four repetitions at the central point was carried out to evaluate the behavior of the electrochemical system assisted by Fenton under different pH levels, dye concentration, and KBPFC concentration. The fixed factors were an agitation of 300 rpm, a current density of 10 mA, and a fixed volume of 70 mL. The values corresponding to each factor are summarized in Table 2, while the specific conditions used in each test can be found in Table 2 and are described in Table 3.

**Table 2:** Independent Variables and Levels Used in the 2^3^ Factorial Design with a Central Point

	–1	0	+1
**KBPFC concentration**	0.2 g/L	0.4 g/L	0.6 g/L
**Dye concentration**	10 ppm	55 ppm	100 ppm
**pH**	3	6	9

**Table 3:** Coded Variables Used in Each Experiment According to the 2^3^ Factorial Design with a Central Point

Experiment	KBPFC concentration	Dye concentration	pH
1	–1	–1	–1
2	+1	–1	–1
3	–1	+1	–1
4	+1	+1	–1
5	–1	–1	+1
6	+1	–1	+1
7	–1	+1	+1
8	+1	+1	+1
9	0	0	0
10	0	0	0
11	0	0	0
12	0	0	0

For the degradation analysis, a Shimadzu UV–Vis Spectrophotometer, model UV-1800, was used. The scanning range was between 200 and 900 nm. To evaluate the degradation rate, eq 4 was used.^15^

4 $$\%removal=\frac{C_{0}-C_{f}}{C_{0}}$$

where *C*_o_ and *C*_f_ are the concentrations before and after the treatment. Data from 2^3^ factorial design with a central point were analyzed using the Design of Experiments package from Origin 2023b (OriginLab Corporation, Northampton, Massachusetts, USA). Decolorization efficiency values used as a response were analyzed using a full quadratic model, yielding coefficients related to the effects of independent variables as well as interaction effects and the lack of fit. The significance of the effects was determined by analysis of variance (ANOVA), taking *p*-values below 0.05 as indicative of significance for both the model obtained and its fit to experimental data.

### 2.7 Kinetic Study

The kinetics of the process were determined using the following *n*th order reaction model:^16^

5 $$\frac{dC}{dt}=-kC^{n}$$

where *C* represents the dye concentration, *n* is the order of the reaction, *k* is the rate constant, and *t* is the time. For the first-order reaction, the equation above, after integration, becomes

6 $$C=C_{0}e^{\left(-kt\right)}$$

where *C*_o_ is the initial dye concentration. For the second-order reaction, the integrated equation is

7 $$C=\frac{C_{0}}{1+kC_{0}t}$$

### 2.8 Total Organic Carbon (TOC) Analysis

The total organic carbon content in the samples was determined with eq 9:^16^

8 $${\%}_{removal}=\frac{TOC_{0}-TOC_{f}}{TOC_{0}}$$

where *TOC*_o_ and *TOC*_f_ are the total organic carbon consumption applied in mg/L after *t* = 0 and *t* = 60 min.

## 3 Results and Discussion

### 3.1 Characterization of the Films

According to ICP-OES analysis, the iron concentration decreased by 15% after the immersion of a 15 g pellicle and by 27% after the immersion of a 20 g pellicle, showing that cation adsorption increases with the increase in BC mass.

The X-ray diffraction (XRD) patterns of the KBP, KBPF, and KBPFC samples reveal significant structural transformations throughout the synthesis process, as shown in Figure 2. The purified bacterial cellulose (KBP) shows two broad peaks centered around 15–25° 2θ, corresponding to the (100), (010), and (110) planes of semicrystalline cellulose, consistent with previous reports.^17^ After iron impregnation (KBPF), the material exhibits additional weak reflections between 30° and 50° 2θ, particularly near 35.5°, 43°, and 57°, which do not match exactly with the standard diffraction profiles of FeCl_2_ or FeCl_3_ (ICSD 01-071-0668 and ICSD 01-077-0999). Instead, these peaks are characteristic of the initial formation of iron oxide species, notably magnetite (Fe_3_O_4_) or maghemite (γ-Fe_2_O_3_), as confirmed by comparison with the ICSD 08-4611 for magnetite.^4,18,19^

**Figure 2:**
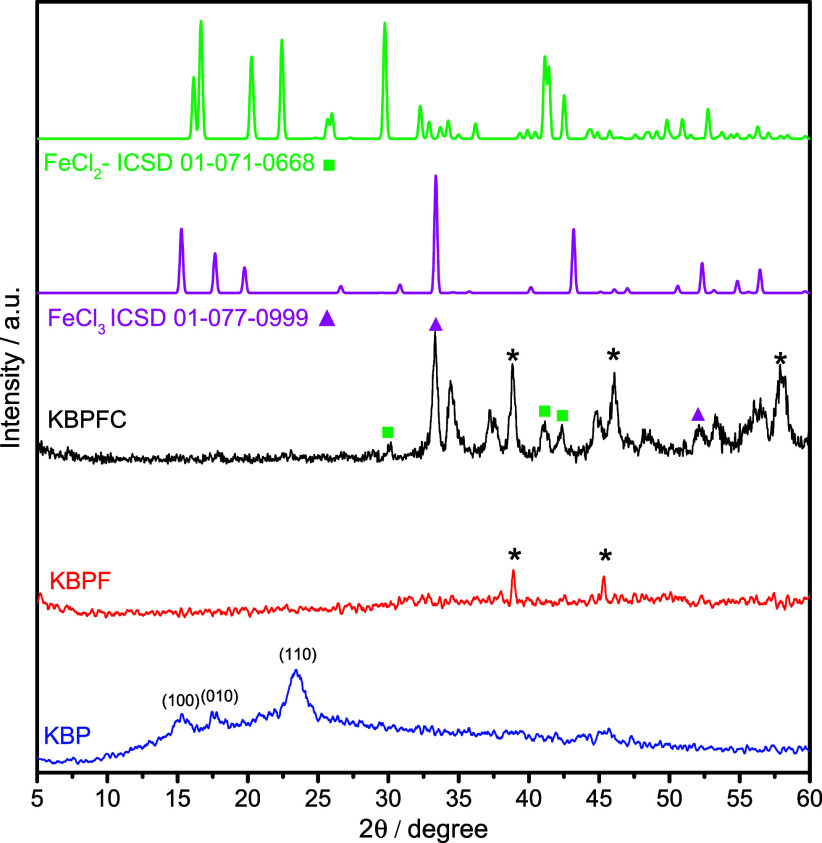
XRD patterns of the KBP, KBPF, and KBPFC samples. Theoretical diffraction peaks of FeCl_3_ are included for comparison.

The reflection near 35.5° corresponds to the (311) plane of magnetite (referring to * in the graph), while the peaks at approximately 43° and 57° match the (400) and (511) planes, respectively. These findings suggest that during the iron impregnation and drying stages, partial oxidation of Fe^2+^/Fe^3+^ occurred, leading to the formation of poorly crystalline or nanocrystalline iron oxides. The low peak intensities and broadening further indicate small crystallite sizes and a high degree of dispersion within the cellulose matrix. Upon calcination (KBPFC), these features become more pronounced, reflecting the stabilization and crystallization of iron oxide phases. This observation confirms that the calcination process facilitates the phase transition from amorphous or poorly ordered intermediates into more crystalline iron oxide structures.

The effect of the different processes to which the bacterial cellulose was subjected was monitored by infrared spectroscopy (Figure 3). Before the purification process, the bacterial cellulose shows a relatively narrow band at 3600 cm^–1^ in addition to a broader one at 3300 cm^–1^, both assigned to OH stretching. The differences in bandwidth probably arise from distinct chemical environments, with the groups responsible for the band at 3600 cm^–1^ having stronger (shorter) and more defined bonds belonging to more ordered regions, while the groups generating the band at 3300 cm^–1^ have slightly weaker bonds and greater participation in hydrogen bonding. The band in the 2905 cm^–1^ region, attributed to CH stretching, becomes more defined after purification. Other changes include the disappearance of the band at 1738 cm^–1^, attributed to C=O groups from biomolecules present in the bacterial structure, along with a clearer definition of the bands at 1658 and 1581 cm^–1^, which are attributed to the angular deformation of H–O–H (water) and C–O–H, as well as the bands at 1434 and 1370 cm^–1^, relating to CH_2_ deformation, and the band corresponding to C–O stretching at 1060 cm^–1^. The CH_2_ deformations mentioned are known as crystallinity bands because they become more defined as the ordering of the cellulose chains increases.^20^ This observation indicates the effectiveness of the purification step.

**Figure 3:**
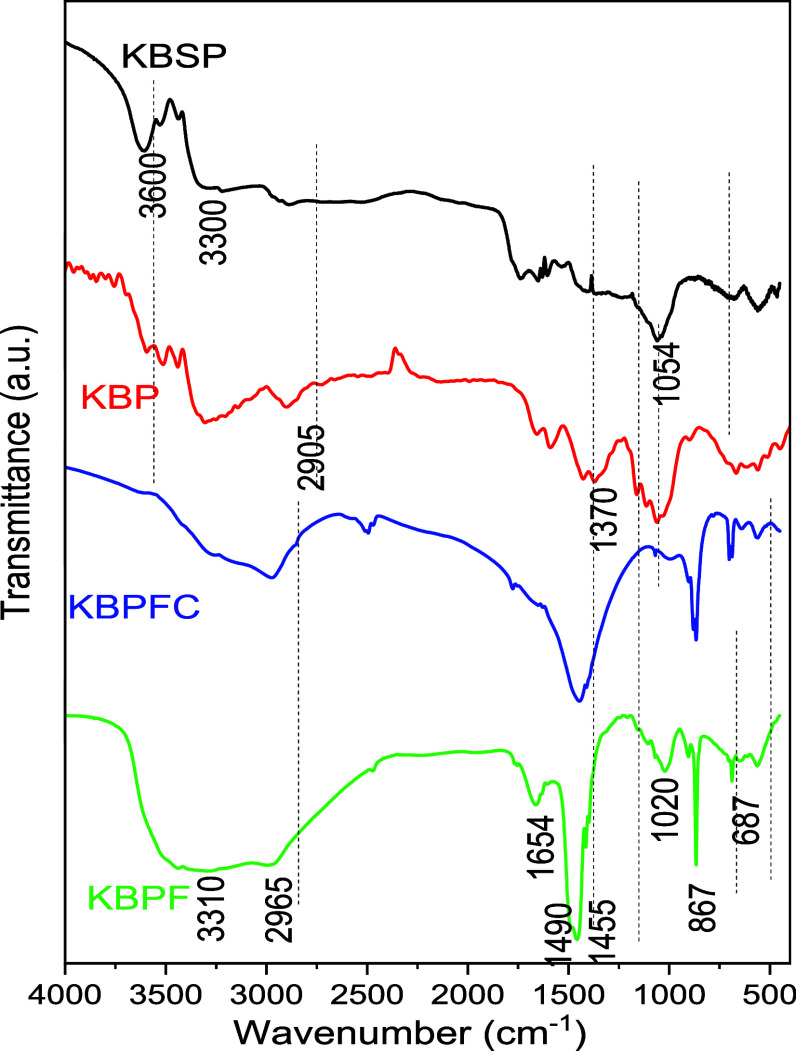
FTIR spectra for the KBSP, KBP, KBPF, and KBPFC samples.

After treatment with Fe^2+^/Fe^3+^, several changes are observed in the infrared spectrum, such as the broadening of the OH stretching band centered at 3310 cm^–1^ and the disappearance of the band at 3600 cm^–1^, indicating that the shorter hydroxyl groups participate in the interaction with the ions. Additionally, a narrow band at 867 cm^–1^ appears, attributed to the asymmetric O–Fe–O stretching,^21^ confirming the modification. Finally, there is also a significant increase in the CH_2_ deformation band at 1455 cm^–1^, which may suggest an increase in the ordering of the chains as this is the so-called crystallinity band. After thermal treatment, a reduction in the OH stretching band at 3300 cm^–1^ occurs, indicating that heating promoted the thermal decomposition of these groups, with an increase in the relative intensity of the CH stretching band at 2956 cm^–1^ and the CH_2_ deformation band at 1448 cm^–1^. This suggests that the oxygenated groups are preferentially removed during thermal decomposition. Finally, the fine band at 867 cm^–1^, attributed to the deformation, remained in the spectrum, indicating the presence of iron-based modifiers.

The TG curves (Figure 4a and 3b) show similar thermal behavior among the uncalcined films, whereas the thermally treated sample exhibits distinct behavior. Up to 110 °C, the mass loss of the films is attributed to the release of physically adsorbed water.^22^ In the KBSP sample, the degradation of low molecular weight compounds from green tea, such as polyphenols and sugars,^23^ occurs between 110 and 300 °C. Above 300 °C, the thermal degradation of CB polymer chains becomes evident, intensifying beyond 400 °C for all films.^23^ Additionally, purification and modification of CB with Fe^2+^/Fe^3+^ resulted in reduced thermal stability, as increased crystallinity and purity of the material enhance thermal conductivity, facilitating heat transfer along the cellulose chains.^24^ The KBPFC sample exhibits notably lower degradation, primarily due to its prior thermal treatment, which had already induced partial decomposition.

**Figure 4:**
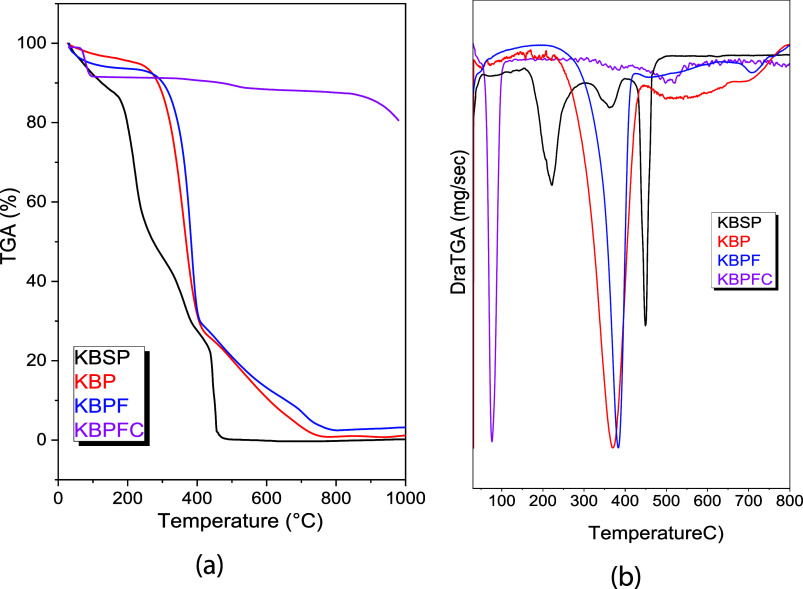
(a) TG curves and (b) DTG curves for KBSP, KBP, KBPF, and KBPFC samples.

The SEM images (Figure 4) reveal significant morphological changes in the samples throughout the preparation process. Before purification (Figure 5a), the film surface displayed oval-shaped bacterial structures along with dispersed fibers. Following NaOH purification (KBP, Figure 5b), an increase in fiber exposure is observed, accompanied by a substantial reduction in visible bacterial structures, which become nearly imperceptible. The deposition of iron oxides (Figure 5c) results in a uniform coating of inorganic particles across the CB surface. After thermal treatment, these particles persist, contributing to the final biochar formation (Figure 5d). In the KBP sample (Figures S2 and S3), the absence of Fe is evident, along with a high concentration of Na arising from the purification process. In contrast, the EDS mapping of the KBPFC film (Figure S4) confirms the homogeneous distribution of Fe and the absence of impurities. Furthermore, the EDS analysis corroborates the presence of Fe in this sample (Figure S5), and the identification of elements and composition through EDX was also verified, as shown in the data in Figure S6.

**Figure 5:**
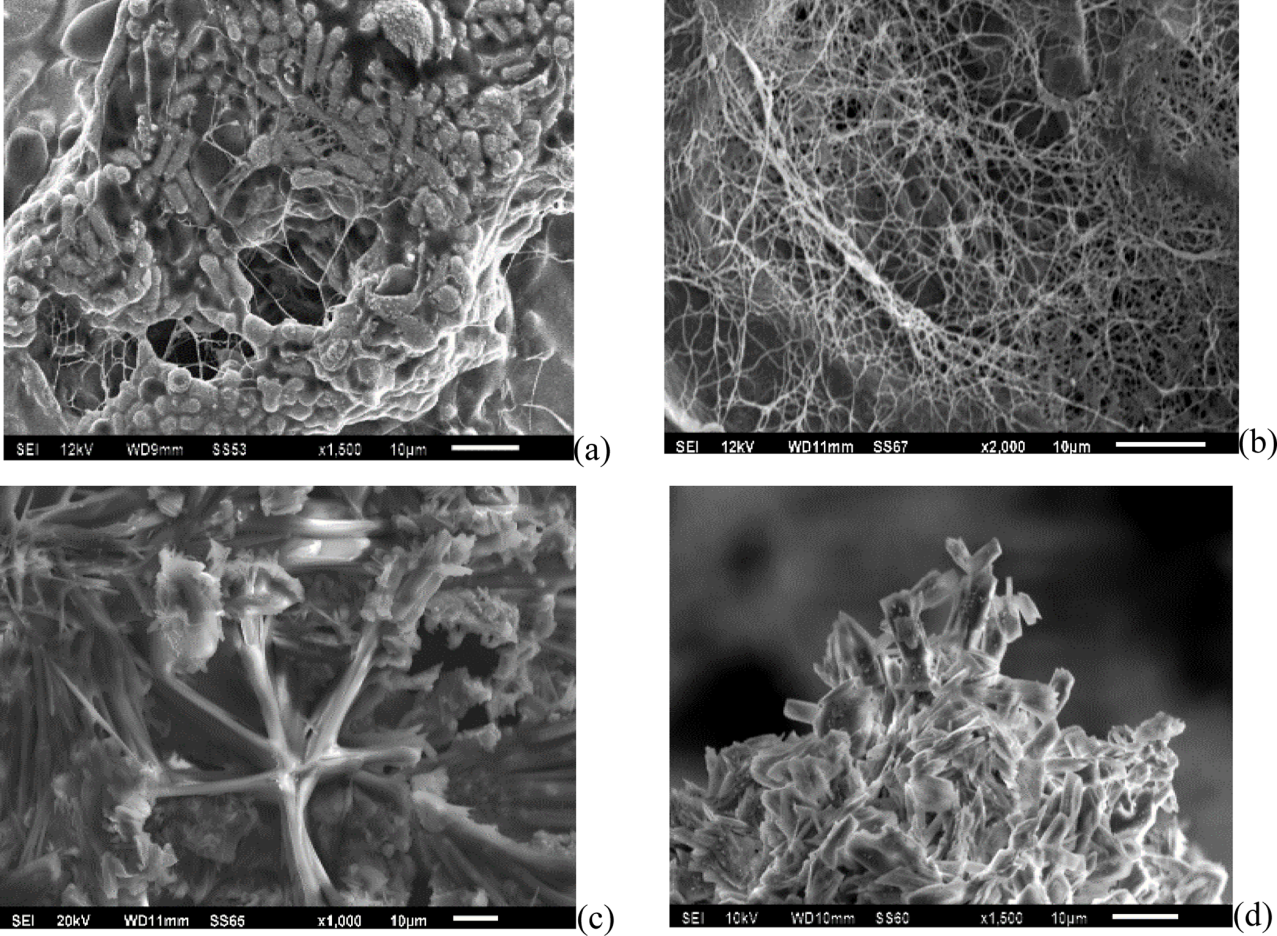
SEM images of the films: (a) KBSP, unpurified bacterial cellulose; (b) KBP, purified with NaOH; (c) KBPF, iron-modified; and (d) KBPFC, iron-modified and thermally treated.

The nitrogen adsorption–desorption isotherms for the KBP, KBPF, and KBPFC samples (Figure S7) exhibit distinct features depending on the treatment applied. According to IUPAC classification, all samples show isotherms with characteristics typical of Type IV profiles, which are associated with mesoporous materials.^25^

The presence of hysteresis loops, particularly in the KBPF and KBPFC samples, indicates capillary condensation within the mesopores. The observed hysteresis resembles Type H3, commonly linked to slit-shaped pores or aggregates of plate-like particles, which is consistent with the layered structure derived from bacterial cellulose and the incorporation of iron species.

The surface area, pore volume, and pore diameter values (Table 4) confirm that calcination significantly enhances the textural properties of the material. KBPFC exhibited the highest surface area (2.811 m^2^/g) and pore volume (0.01820 cm^3^/g), with an average pore diameter remaining within the mesoporous range (2–50 nm).^26,27^ These properties are advantageous for catalytic applications, promoting better accessibility and dispersion of the reactive sites. The nitrogen adsorption–desorption isotherms and pore volume distributions obtained by the DFT method can be seen in Figure S7.

**Table 4:** Surface Characteristics of KBP, KBPF, and KBPFC Samples

Sample	KBP	KBPF	KBPFC
**Surface area (m** ^**2**^ **/g)**	0.479	0.789	2.811
**Pore volume (cm** ^**3**^ **/g)**	0.00104	0.00150	0.01820
**Pore diameter (nm)**	1.7339	2.2713	2.0758

### 3.2 Degradation Analysis in Samples Containing MB

#### 3.2.1 Initial Studies

To assess the impact of KBPFC-Fenton on the electrochemical degradation of methylene blue (MB), experiments were conducted using 10 ppm solutions under three conditions: electrochemical degradation, a heterogeneous Fenton process, and their combined effect. The experiments were performed at pH 6, with a KBPFC concentration of 0.4 g/L in the Fenton systems. The discoloration values for all experiments are listed in Table S1. After 60 min, the electrochemical system achieved a discoloration rate of 62.74%, while the heterogeneous Fenton process reached 60.09%. When both treatments were applied simultaneously, the degradation rate increased to 81.42%, representing nearly a 20% enhancement compared with the isolated techniques. This improvement is likely due to the intensified generation of reactive species, including hydroxyl radicals and Fe^2+^. Given this superior performance, the electrochemically assisted heterogeneous Fenton process was selected for further optimization through a design of experiments, as outlined in the following section.

The effect of H_2_O_2_ concentration in batch Fenton was evaluated through the addition of 1, 3, and 5 drops of H_2_O_2_ 35% (Figure S8), revealing that a 5-fold increase in H_2_O concentration resulted in a change from around 68% to 77% degradation as shown in the figure below. Thus, even the lowest concentration evaluated, related to the addition of a single drop, is sufficient to provide a satisfactory degradation degree. Also, the evaluation of radical species was carried out through the use of scavengers, including sodium azide (singlet oxygen scavenger), *tert*-butyl alcohol, and potassium iodide that scavenge hydroxyl radicals from the bulk solution and at the catalyst surface, respectively (Figure S9). This revealed that the most significant decrease in degradation efficiency occurred in the presence of potassium iodide, indicating that the most important radical species are hydroxyl radicals present at the catalyst surface.

#### 3.2.2 Factorial Design 2^3^ with a Central Point

The 2^3^ factorial design with four repetitions of the central point was carried out in order to evaluate the effect of variables used in the electrochemically assisted Fenton process. The data treatment using a full quadratic model (Table 5) showed that the effect with the largest magnitude was the dye concentration, whose increase negatively impacted the degradation percentage. The second most important effect in magnitude was the biochar concentration (KBPFC), which positively influenced the degradation percentage. Only these two effects were statistically significant, as the statistical analysis of results (Table 4 and the Pareto chart shown in Figure 5a) showed that the effect of pH was statistically insignificant. The time evolution of MB concentration and degradation efficiency for different tests (Figure 6b,c) evidenced that changes in MB concentration were primarily influenced by its initial value, while decolorization efficiency was influenced by both dye and biochar concentrations. For instance, Figure 5b displays three distinct groups of curves, each corresponding to one of the three initial MB concentrations tested. In contrast, Figure 5c illustrates four groups of curves according to decreasing decolorization efficiency.

**Table 5:** Regression Coefficients and Analysis of the Model

	Effect
**pH**	2 ± 1.395
**[dye]**	-58.15 ± 1.395
**[calcined]**	9.65 ± 1.395
pH*[dye]	1.35 ± 1.395
pH*[calcined]	-3.45 ± 1.395
[dye]*[calcined]	4.2 ± 1.395
**Degrees of freedom**	5
**Root-mean-square error**	3.94485
**R-square**	*R*^2^ = 0.989

**Figure 6:**
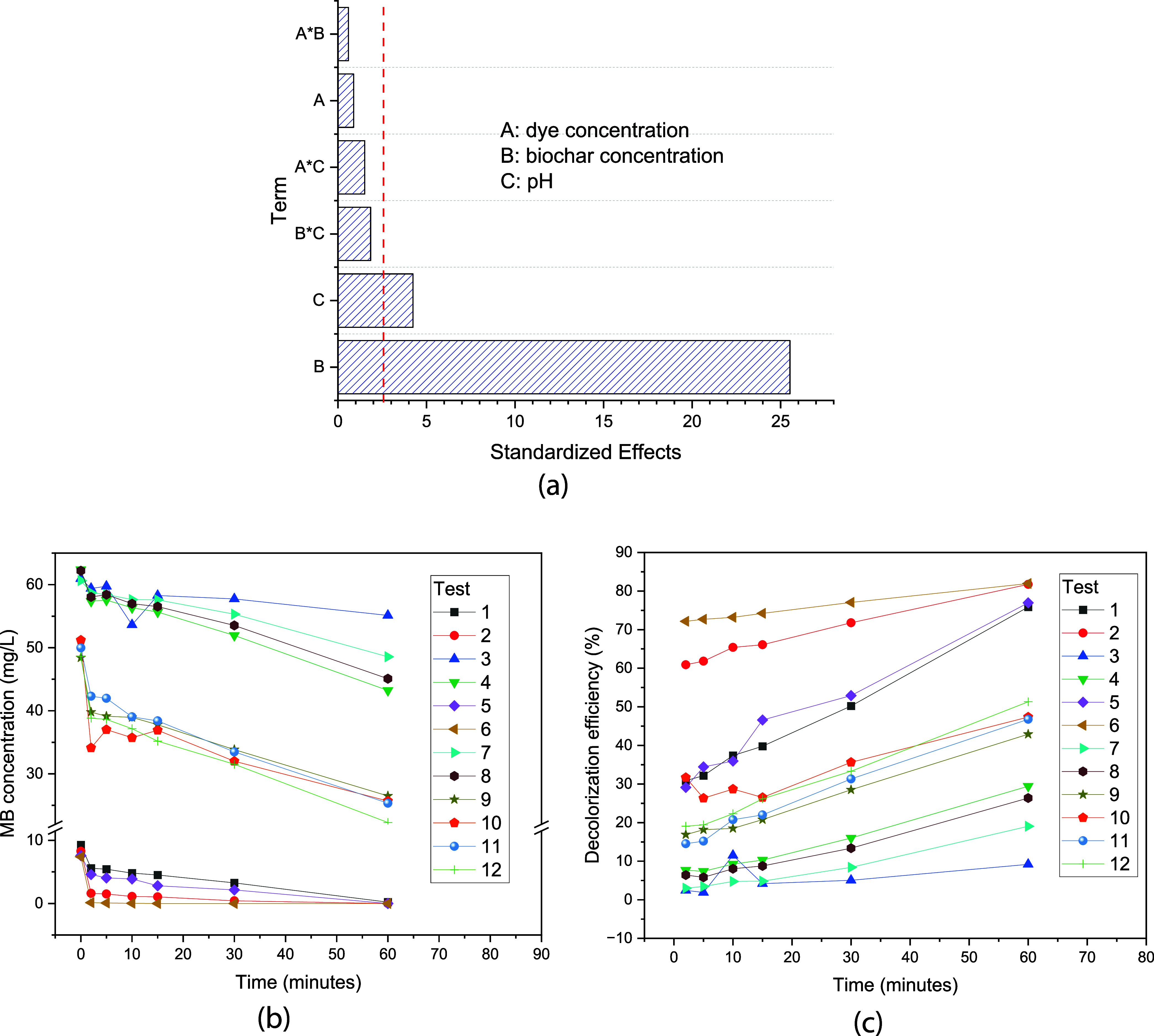
(a) Pareto chart illustrating the relative significance of variables influencing the decolorization process; (b) methylene blue (MB) concentration profiles across the experimental runs; (c) percentage of decolorization achieved in each factorial design experiment at various treatment times.

The highest decolorization efficiencies were observed in tests 6 and 2, which corresponded to the lowest dye concentration (level −1) and the highest biochar concentration (level +1). The next group involved tests 1 and 5 and exhibited slightly lower efficiency, as both dye and biochar concentrations were set to their lowest levels (level −1). This was followed by four replicates of the central point, which demonstrated intermediate efficiency. The lowest decolorization efficiency was observed in tests 4, 8, 7, and 3, where the MB concentration was at the highest level (level +1) and the biochar concentration at its lowest (level −1). These results highlight the critical role of both dye and biochar concentrations in determining the efficiency of the decolorization process. Furthermore, although the interaction effect between dye and calcined BC concentrations was more pronounced than the pH effect, it was not statistically significant. This suggests that under the tested conditions, the combined influence of these variables did not substantially impact the overall process efficiency. Additionally, it is possible to verify in Table 6 the ANOVA performed on decolorization percentages as outlined in the experimental design.

**Table 6:** Analysis of Variance (ANOVA) of the Percentage of Decolorization According to the Design of Experiments

	Degrees of freedom	Sum of squares	Mean square	*F*-value	Prob > *F*
**pH**	1	8	8	0.514	0.505
**[dye]**	1	6762.845	6762.845	434.579	4.4 × 10^–6^
**[calcined]**	1	186.245	186.245	11.968	0.018
pH*[dye]	1	3.645	3.645	0.234	0.649
pH*[calcined]	1	23.805	23.805	1.530	0.271
**Error**	5	77.80917	15.5618		
**total**	11	7097.62917			

Regarding the results of different experiments (Figure 6b), the data varied significantly depending on the variables, with the best degradation rate at 10 ppm obtained from experiment 6, although it is very close to the result of experiment 2. Both experiments used a KBPFC concentration of 0.6 g/L and differed only in the pH of the medium, showing the advantage of the heterogeneous Fenton process regarding its applicability independent of pH.

### 3.3 Kinetic Study

The study of the reaction kinetics (Figure 6) was conducted using data from the 4 central point experiments. The second-order kinetics model (Figure 7b) showed a poorer fit compared to the first-order model (Figure 7a), as indicated by the higher regression coefficient (>0.98) for the latter. The best fit followed first-order kinetics, with a rate constant of *k* = 0.0809 s^– 1^ and a half-life calculated as *t*_1/2_ = ln2/*k*, resulting in *t*_1/2_ = 8.6 min. Wang et al.^16^ reported that several researchers found that the Fenton reaction for dye degradation follows pseudo-first-order kinetics, while the author, when considering the entire degradation rate of the process, observed a combination of first-order (Fe^3+^/H_2_O_2_) and second-order (Fe^2+^/H_2_O_2_) kinetics for the treatment with the dye Acid Black C.I.

**Figure 7:**
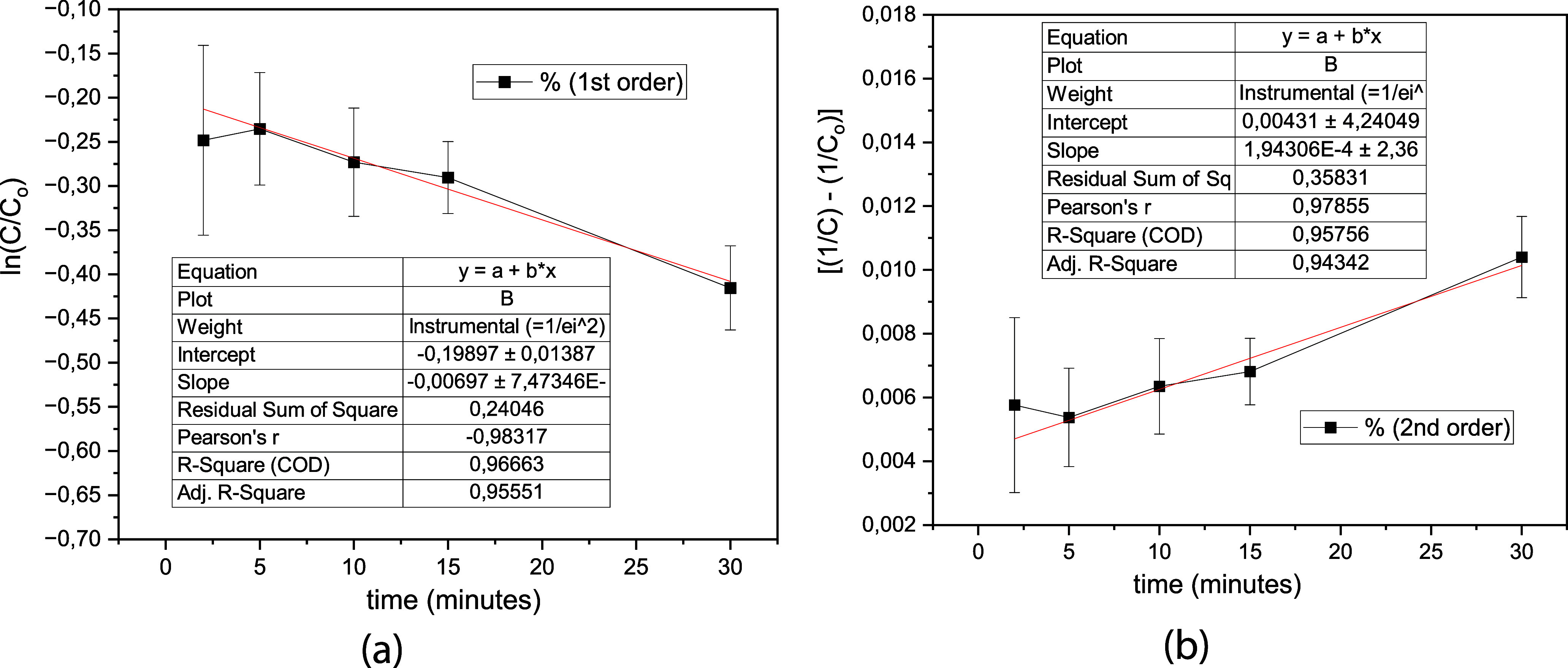
(a) First- and (b) second-order kinetic models for the degradation of MB at 55 ppm.

### 3.4 Total Organic Carbon Analysis (TOC)

The TOC analysis revealed a percentage reduction in total organic carbon for different initial MB concentrations: 56.10% for 10 ppm, 51.21% for 55 ppm, and 19.52% for 100 ppm. These results confirm the trend observed in the spectrophotometric analysis, indicating that decolorization significantly contributes to mineralization rather than merely causing structural modifications of the chromophore group. Watwe, Kulkarni, and Kulkarni^28^ achieved a 61% TOC reduction within 60 min using a Fenton-like oxidation process by replacing Fe­(II) with Cr­(VI), but for MB concentrations of approximately 5.0 ppm (15.7 μM). Similarly, Kirchon et al.^29^ reported a 31% TOC reduction after 120 min using iron-based metal–organic frameworks in a Fenton and photo-Fenton process for 15 ppm MB, with optimal results requiring up to 720 min. In contrast, Cao et al.^30^ attained a 77.30% TOC reduction within just 10 min but for a lower MB concentration of 6.4 ppm. Comparing these findings with the literature data suggests that the results obtained in this study are highly competitive. Further optimization, such as extending reaction times and fine-tuning material dosages, could potentially enhance overall efficiency and mineralization rates.

Compared to previous studies, the system proposed in this work is among those with efficiencies higher than 80% and ranks among the three fastest processes reported to date. This highlights its competitive performance, despite being based on low-cost and readily available materials, as summarized in Table 7.

**Table 7:** Comparison of Performance and Conditions of This Work and Literature Data

Reference	Process	Catalyst	Dye	Concentration	Removal (%)	Time (min)
Wibowo et al (2018)^4^	Heterogeneous Fenton	Fenton catalyst@BC aerogels	Methylene blue	3 ppm	∼80	120
Santana et al. (2023)^1^	Photo-Fenton/sunlight/BC/Fe_3_O_4_	BC/Fe_3_O_4_ membranes	Mixture of four textile dyes, including methylene blue	50 ppm	99	180
Watwe, Kulkarni, and Kulkarni (2021)^28^	Homogeneus Fenton oxidation	Without catalyst, only replacing Fe(II) with Cr(VI)	Methylene blue	5 ppm	99%	30
Lin et al. (2024)^31^	Photo-Fenton reactions	Mn_3_O_4_/Mg(OH)_2_-doped activated carbon	Methylene blue	10 ppm	88%	180
Zhang et al. (2024)^32^	Photovoltaic (PV)-powered electro-Fenton (EF) technology	Addition of Fe^2+^ as a catalyst	Methylene blue	20 ppm	81%	20
Khudkham et al. (2022)^33^	Photo-Fenton-like catalyst	Cu(II)–quinoline complex immobilized on a silica support	Methylene blue	5 ppm	95%	150
Kirchon et al. (2020)^29^	Fenton and photo-Fenton reactions	Isomorphic substitution of Mn and Co for Fe	Methylene blue	15 ppm	100%	300
This work	Electrochemically assisted heterogeneous Fenton	KBPFC	Methylene blue	10 ppm	∼82%	60

In summary, this study demonstrates the potential of biochars derived from modified bacterial cellulose (KBPFC) as effective catalysts for MB dye degradation, particularly when integrated with heterogeneous Fenton and electrochemical systems. The factorial design enabled the optimization of key operational parameters such as dye concentration and pH. The highest decolorization efficiency, exceeding 80%, was achieved using 0.6 g/L KBPFC for 10 ppm MB solutions, with promising results also observed at 55 ppm. Additionally, a significant reduction in the TOC was achieved, indicating effective mineralization. The degradation kinetics followed a pseudo-first-order model, with a rate constant (*k*) of 0.0809 s^– 1^, reinforcing the efficiency of the process. However, the performance declined with increasing dye concentration, suggesting that pollutant load plays a critical role in system effectiveness. The combination of electrochemical treatment with the heterogeneous Fenton process proved advantageous, enhancing the degradation rate and demonstrating the synergistic benefits of this approach.

## Supplementary Material



## Notes

Schematic of the electrochemical system used for methylene blue degradation (Figure S1), EDS mapping of the KBP film (Figure S2), elemental composition plot of the KBP film by EDS (Figure S3), EDS mapping of the KBPFC film (Figure S4), elemental composition plot of the KBPFC film by EDS (Figure S5), comparative EDX elemental mapping of KBP, KBPF, and KBPFC samples (Figure S6), nitrogen adsorption–desorption isotherms and DFT pore size distributions of KBP, KBPF, and KBPFC (Figure S7), dependence of degradation efficiency on the number of H_2_O_2_ 35% drops (Figure S8), the effect of radical scavengers on degradation performance (Figure S9), and a table with the percentage of discoloration obtained in each factorial design experiment (Table S1).(PDF)

## Abbreviations

BC: Bacterial cellulose
MB: Methylene blue
SEM/EDS: Scanning electron microscopy and energy-dispersive X-ray spectroscopy
