# Supplementary material for: Bacterial Cellulose-Derived Biochar for Electrochemically Assisted Fenton Degradation of Methylene Blue
Source: ACS Omega. 2025 Jun 16;10(25):26953–63. doi: 10.1021/acsomega.5c02022 (PMC12223840; doi:10.1021/acsomega.5c02022)
Supplement: Supplementary file 1 [file ao5c02022_si_001.pdf]

Supplementary information for the paper

Bacterial cellulose-derived biochar for electrochemically-assisted Fenton degradation of methylene blue

Gladston L. dos Santos[1], Rebeca E. S. Barros[2,3], Yslaine A. de Almeida[1], Katlin I. B. Eguiluz[2,3], Giancarlo R. Salazar-Banda[2,3], and Iara F. Gimenez[1]*

[1] Programa de Pós-graduação em Química, Universidade Federal de Sergipe, 49100-000, São Cristovão, SE, Brazil

[2] Laboratório de Eletroquímica e Nanotecnologia – LEN, Instituto de Tecnologia e Pesquisa – ITP, 49032–490, Aracaju, Sergipe, Brazil

[3] Programa de Pós-graduação em Engenharia de Processos, Universidade Tiradentes – UNIT, 49032–490, Aracaju, Sergipe, Brazil

*Corresponding author. E-mail: gimenez@academico.ufs.br

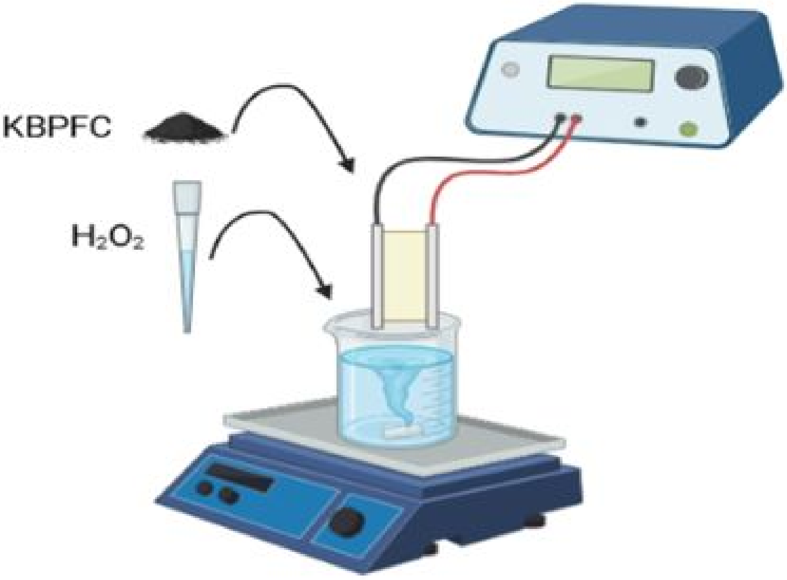

Figure S1. Schematic of the electrochemical system used to treat MB.

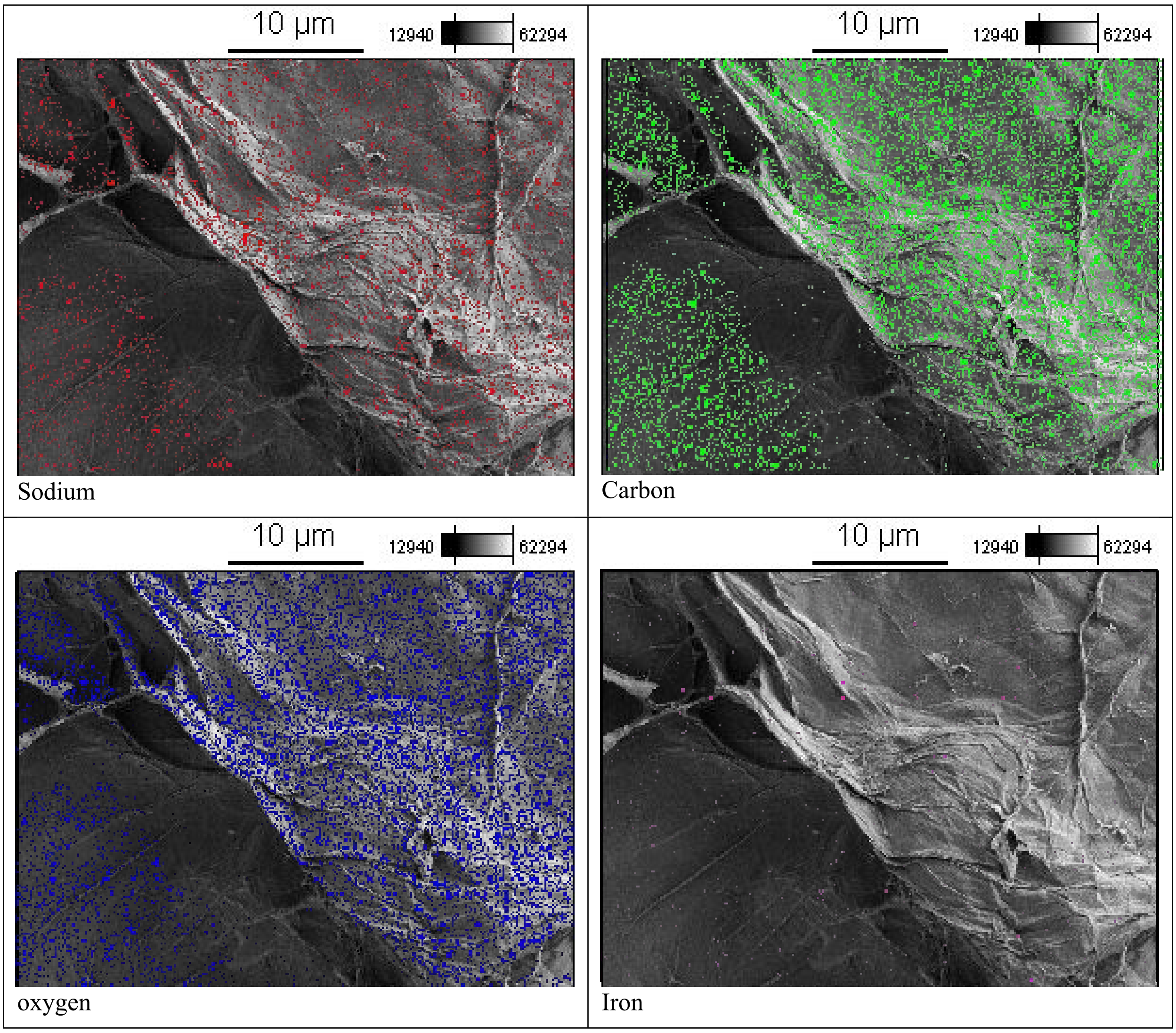

Figure S2. KBP film mapping via energy dispersive spectroscopy.

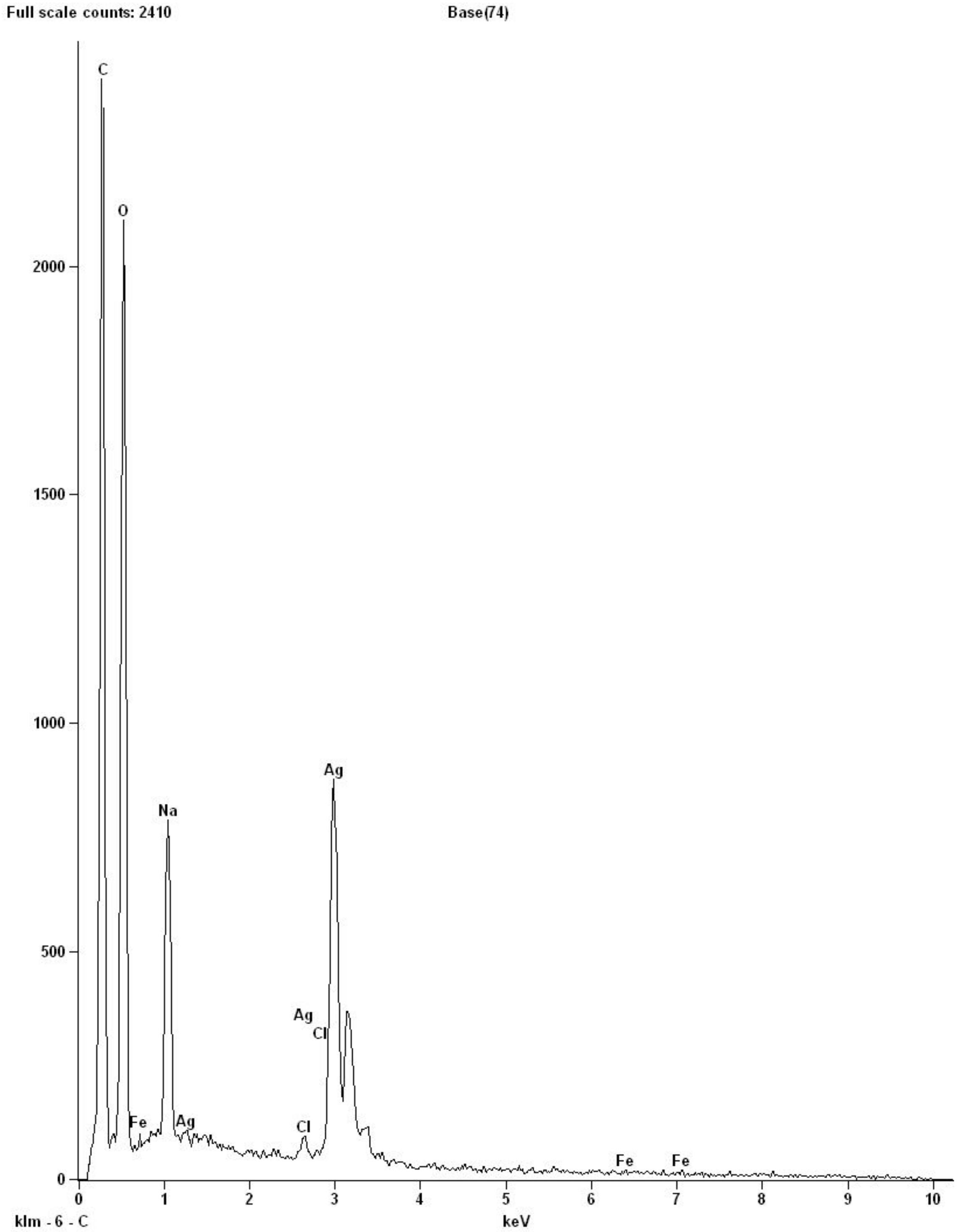

Figure S3. Element count plot of KBP film using energy dispersive X-ray spectroscopy.

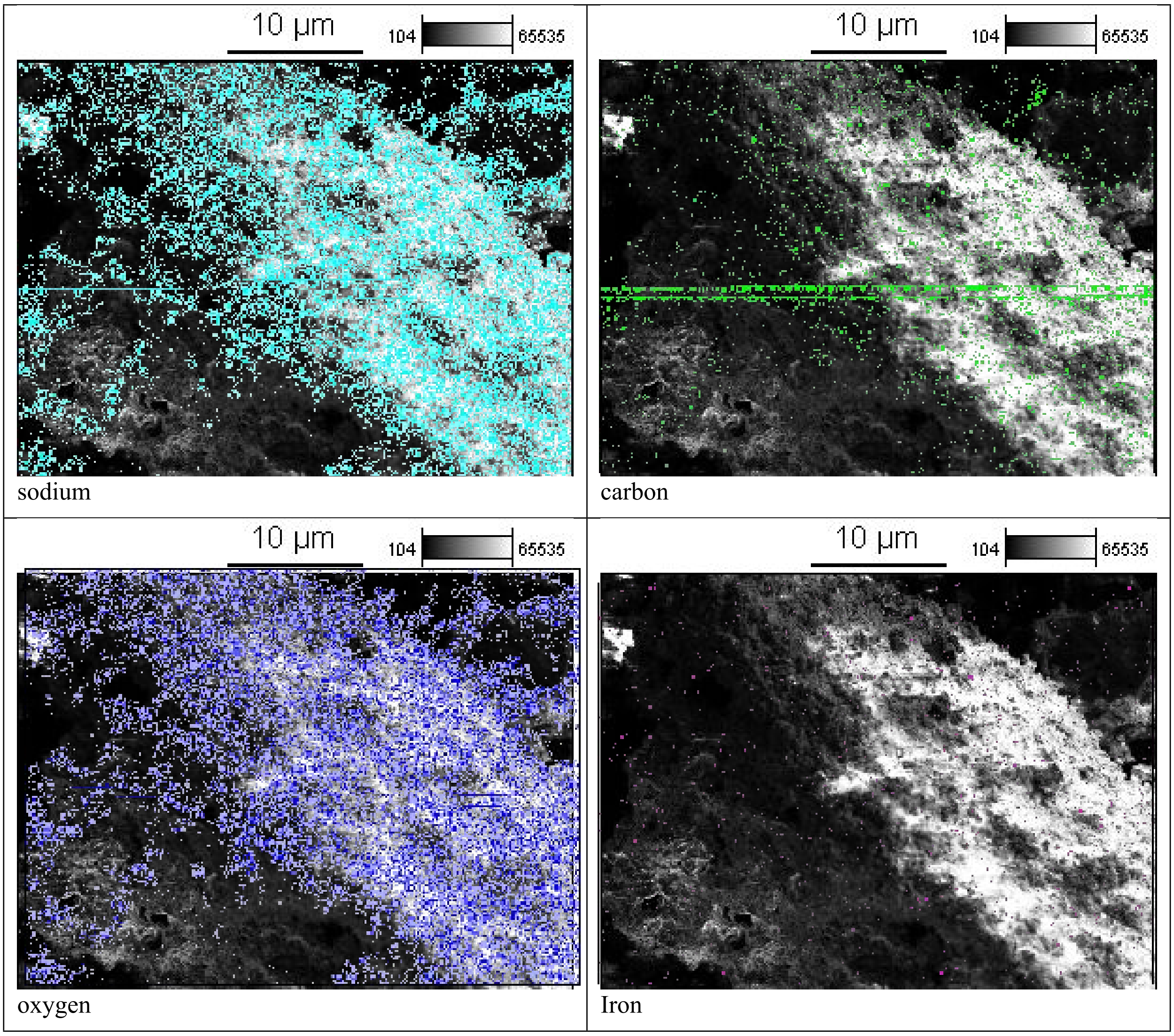

Figure S4. KBPFC film mapping via energy dispersive spectroscopy.

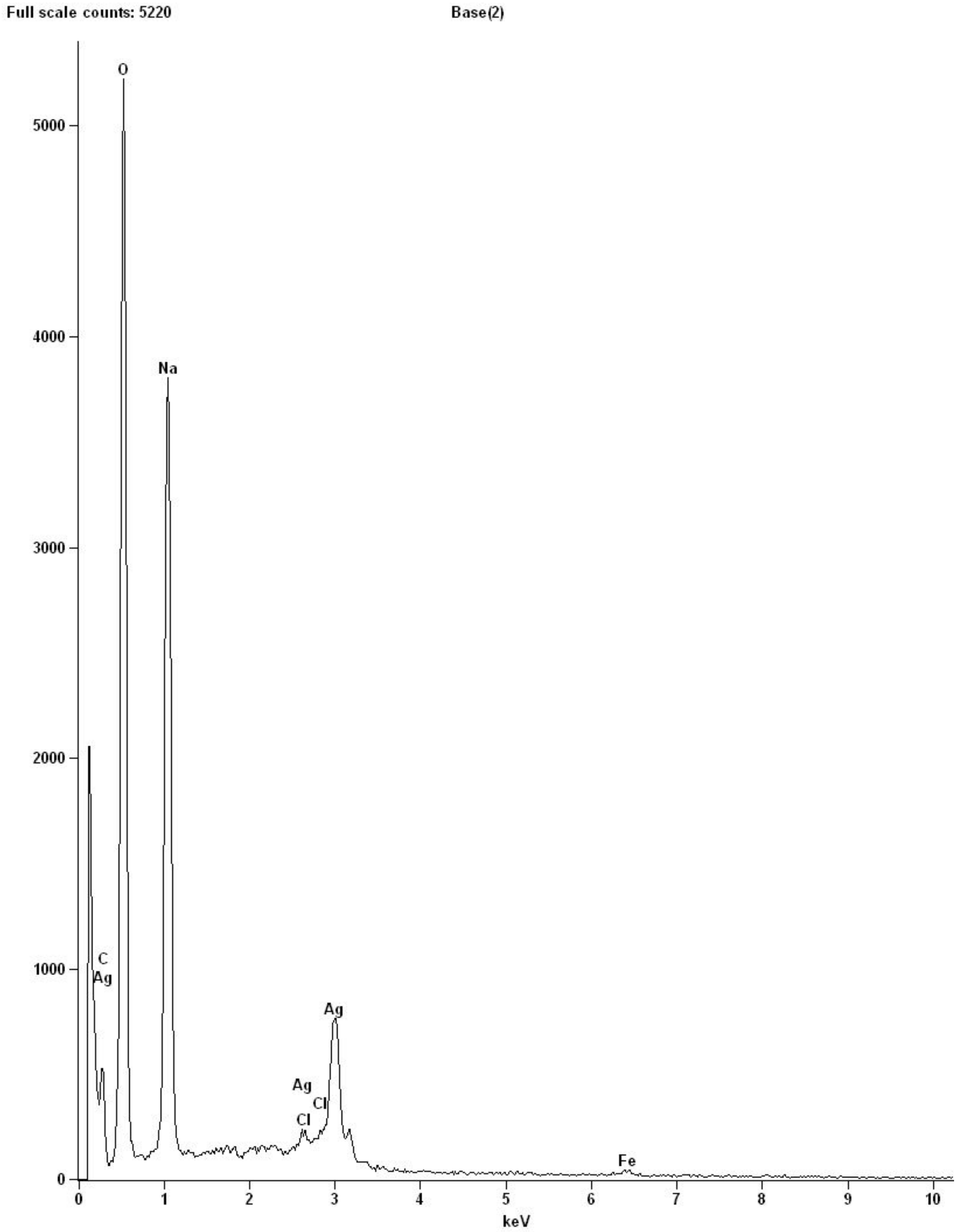

Figure S5. Element count plot of KBPFC film using energy dispersive X-ray spectroscopy.

Sample : KBP

Analyte	Result		[3-sigma]	Proc.-Calc.	Line	Intensity
Ca	0.031	%	[0.000]	Quant.-FP	CaKa	0.3108
S	0.016	%	[0.001]	Quant.-FP	S Ka	0.0279
Cl	0.014	%	[0.003]	Quant.-FP	ClKa	0.0042
K	0.004	%	[0.000]	Quant.-FP	K Ka	0.0549
Fe	0.001	%	[0.000]	Quant.-FP	FeKa	0.1328
Ni	0.000	%	[0.000]	Quant.-FP	NiKa	0.1156
Cu	0.000	%	[0.000]	Quant.-FP	CuKa	0.1200
Zn	0.000	%	[0.000]	Quant.-FP	ZnKa	0.0043
C	99.933	%	[------]	Balance	--------	--------

Sample : KBPF

Analyte	Result		[3-sigma]	Proc.-Calc.	Line	Intensity
Fe	0.580	%	[0.002]	Quant.-FP	FeKa	65.1640
Cl	0.065	%	[0.004]	Quant.-FP	ClKa	0.0193
Ca	0.015	%	[0.001]	Quant.-FP	CaKa	0.1495
Mn	0.001	%	[0.000]	Quant.-FP	MnKa	0.0900
Cu	0.000	%	[0.000]	Quant.-FP	CuKa	0.2306
Zn	0.000	%	[0.000]	Quant.-FP	ZnKa	0.0074
C	99.337	%	[------]	Balance	--------	--------

Sample : KBPFC

Analyte	Result		[3-sigma]	Proc.-Calc.	Line	Intensity
Fe	0.401	%	[0.002]	Quant.-FP	FeKa	45.6693
Cl	0.119	%	[0.006]	Quant.-FP	ClKa	0.0353
Ca	0.011	%	[0.001]	Quant.-FP	CaKa	0.1061
K	0.009	%	[0.001]	Quant.-FP	K Ka	0.0175
Cu	0.001	%	[0.000]	Quant.-FP	CuKa	0.3595
C	99.459	%	[------]	Balance	--------	--------

Figure S6: Mapping of elements present in KBP, KBPF, and KBPFC samples via energy dispersive X-ray spectroscopy.

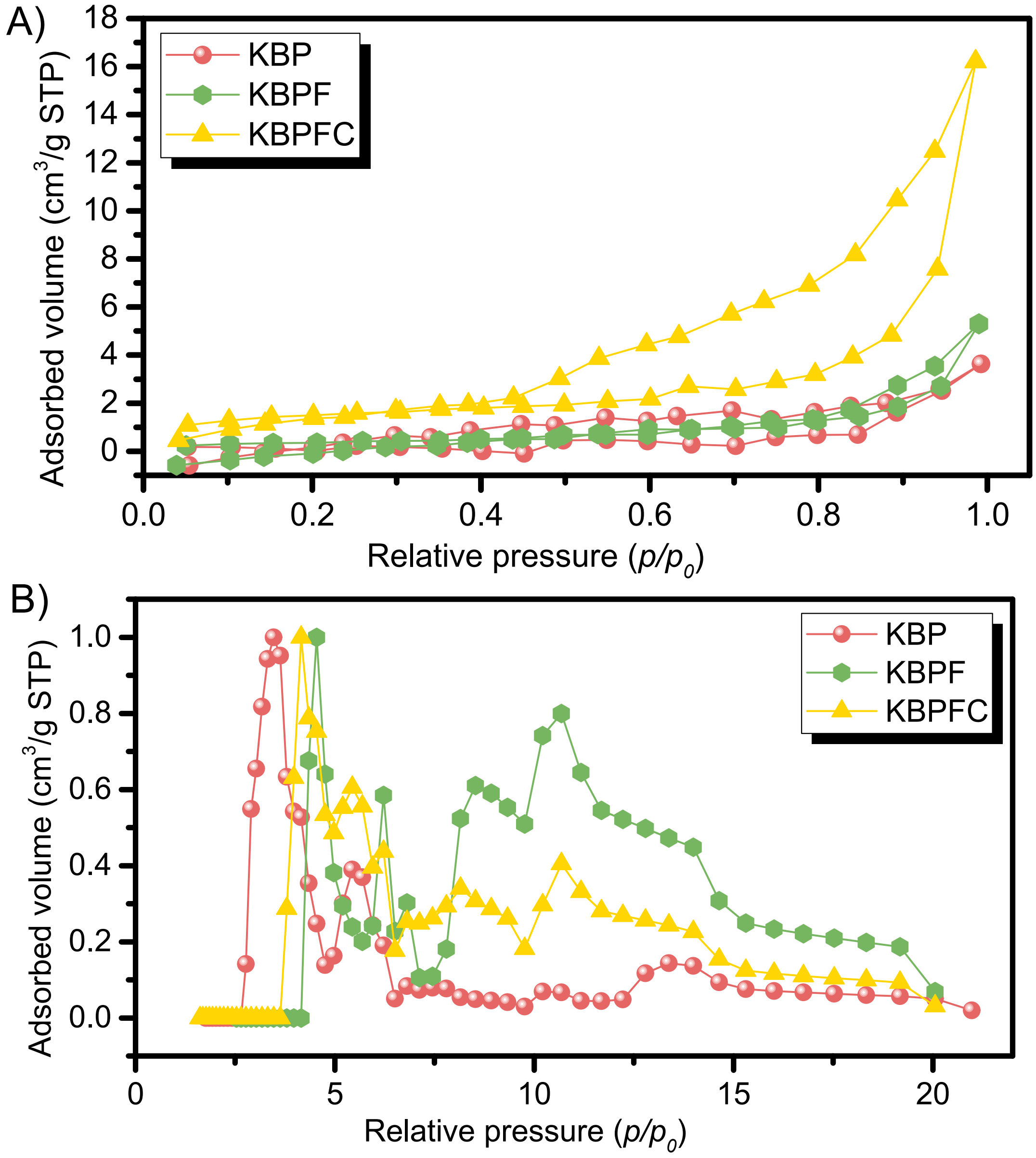

Figure S7: Nitrogen adsorption-desorption isotherms (A) and pore volume distributions obtained by the DFT method (B) for the bacterial cellulose derived from KBP, KBPF, and KBPFC samples.

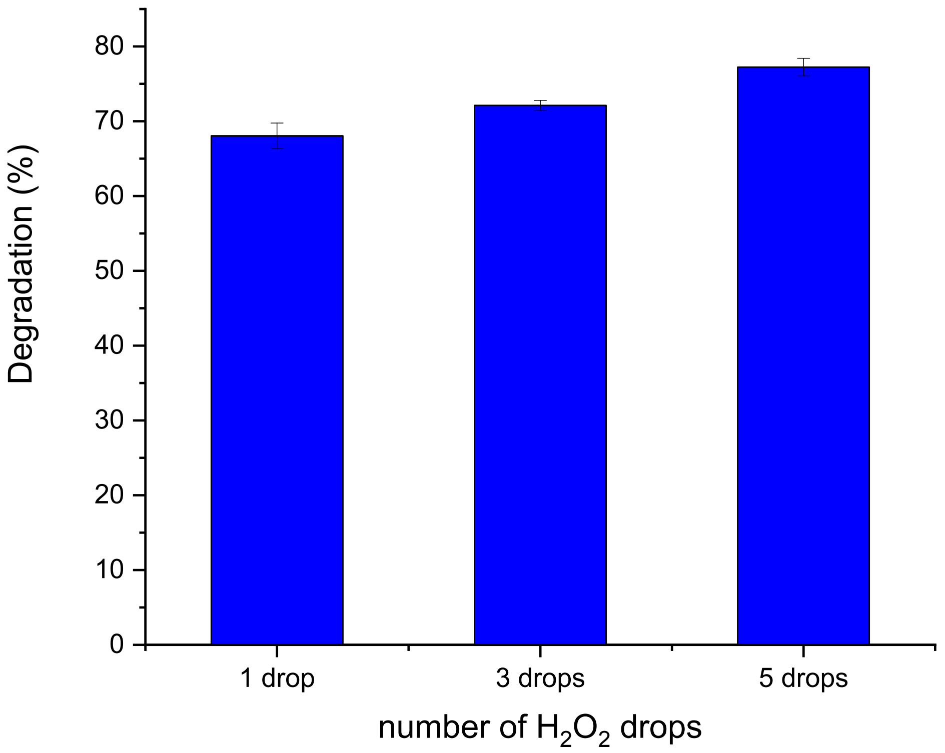

Figure S8: Effect of the number of 35% H_2O_2 drops on the percentage of degradation.

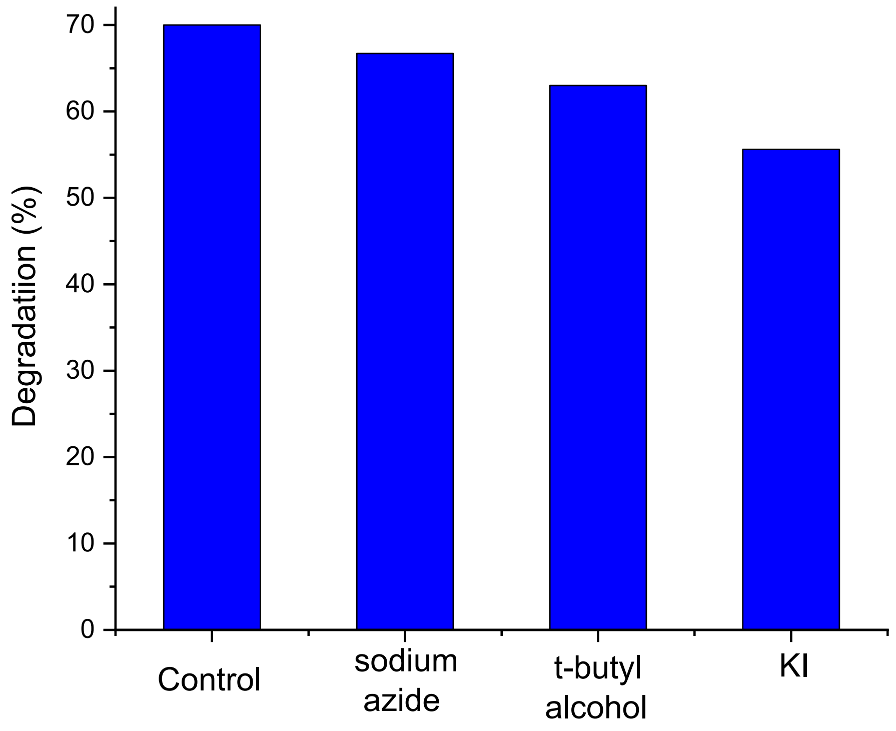

Figure S9. Percentage of degradation observed in the absence and presence of radical scavengers.

Table S1. Percentage of discoloration for each experiment of the factorial design

Experiment	% degradation
1	75.87
2	81.75
3	9.18
4	29.52
5	76.96
6	81.96
7	19.07
8	26.38
9	42.91
10	47.35
11	46.77
12	51.34
